# Human Amniotic Epithelial Cell Transplantation Induces Markers of Alternative Macrophage Activation and Reduces Established Hepatic Fibrosis

**DOI:** 10.1371/journal.pone.0038631

**Published:** 2012-06-14

**Authors:** Ursula Manuelpillai, Dinushka Lourensz, Vijesh Vaghjiani, Jorge Tchongue, Derek Lacey, Jing-Yang Tee, Padma Murthi, James Chan, Alexander Hodge, William Sievert

**Affiliations:** 1 Center for Reproduction and Development, Monash Institute of Medical Research, Monash University, Melbourne, Australia; 2 Center for Inflammatory Diseases, Monash University, Melbourne, Australia; 3 University of Melbourne, Arthritis and Inflammation Research Centre, Royal Melbourne Hospital, Melbourne, Australia; 4 Department of Obstetrics and Gynecology, University of Melbourne, Melbourne, Australia; 5 Gastroenterology and Hepatology Unit, Southern Health, Melbourne, Australia; 6 Pregnancy Research Center, Department of Perinatal Medicine, Royal Women’s Hospital, Melbourne, Australia; National Institutes of Health, United States of America

## Abstract

Chronic hepatic inflammation from multiple etiologies leads to a fibrogenic response that can progress to cirrhosis and liver failure. Transplantation of human amniotic epithelial cells (hAEC) from term delivered placenta has been shown to decrease mild to moderate hepatic fibrosis in a murine model. To model advanced human liver disease and assess the efficacy of hAEC therapy, we transplanted hAEC in mice with advanced hepatic fibrosis. Immunocompetent C57BL/6 mice were administered carbon tetrachloride (CCl_4_) twice weekly resulting in bridging fibrosis by 12 weeks. hAEC (2×10^6^) were infused via the tail vein at week 8 or weeks 8 and 10 (single and double dose, respectively). Human cells were detected in mouse liver four weeks after transplantation showing hAEC engraftment. CCl_4_ treated mice receiving single or double hAEC doses showed a significant but similar decrease in liver fibrosis area associated with decreased activation of collagen-producing hepatic stellate cells and decreased hepatic protein levels of the pro-fibrogenic cytokine, transforming growth factor-beta1. CCl_4_ administration caused hepatic T cell infiltration that decreased significantly following hAEC transplantation. Hepatic macrophages play a crucial role in both fibrogenesis and fibrosis resolution. Mice exposed to CCl_4_ demonstrated increased numbers of hepatic macrophages compared to normal mice; the number of macrophages decreased significantly in CCl_4_ treated mice given hAEC. These mice had significantly lower hepatic protein levels of the chemokine monocyte chemoattractant protein-1 than mice given CCl_4_ alone. Alternatively activated M2 macrophages are associated with fibrosis resolution. CCl_4_ treated mice given hAEC showed increased expression of genes associated with M2 macrophages including YM-1, IL-10 and CD206. We provide novel data showing that hAEC transplantation induces a wound healing M2 macrophage phenotype associated with reduction of established hepatic fibrosis that justifies further investigation of this potential cell-based therapy for advanced hepatic fibrosis.

## Introduction

Chronic hepatic inflammation from diverse causes including alcohol, steatohepatitis, autoimmune disease and viral infection leads to a wound healing, pro-fibrogenic response. In some patients with ongoing liver injury, this response can progress to cirrhosis, portal hypertension and liver failure [1]. These outcomes are associated with a significant mortality rate for which liver transplantation is the only curative therapy [2], [3]. However, low donor numbers, high procedural costs and the requirement for life-long immunosuppression limit the number of patients who undergo transplantation and consequently alternative therapies have been sought. Among these, the transplantation of hematopoietic and mesenchymal stem cells derived from adult bone marrow and placenta have shown beneficial effects in animal models of hepatic fibrosis [4], [5], [6], [7] leading to early phase clinical trials using autologous bone marrow derived cells [8], [9], [10], [11]. Most of the clinical trials have been small, often with less than ten patients, and uncontrolled but have shown short-term clinical benefits [12]. Recently, we have shown that transplantation of placenta derived human amniotic epithelial cells (hAEC) into immunocompetent mice with carbon tetrachloride (CCl_4_) induced liver fibrosis can constrain hepatic fibrogenesis [13]. This outcome may be related to several factors linked to hAEC transplantation including reduction in the expression of pro-inflammatory and pro-fibrogenic cytokines coupled with the induction of matrix metalloproteinases to promote a collagen-degrading environment [13].

During pregnancy, hAEC form a monolayer lining the inner of two membranes retaining the amniotic fluid surrounding the fetus. Unlike adult bone marrow derived stem cells, hAEC are highly abundant and easily harvested from term delivered amnion membranes typically yielding over 150×10^6^ cells/membrane and thereby minimizing the need for expensive and time consuming cell expansion [14]. hAEC are derived from embryonic epiblast cells prior to gastrulation and possess some features of their founder pluripotent stem cells including the ability to differentiate into multiple lineages derived from the primary germ layers [14], [15]. Importantly, like other fetal-derived placental cells that evade maternal immune recognition and secrete factors known to dampen maternal immune responses against the fetal semi-allograft, hAEC have also been shown to have low immunogenicity and the capacity to modulate innate and adaptive immune cell responses [14], [16], [17]. Collectively, these features make hAEC an attractive source of cells for potential therapeutic applications.

While we have shown ameliorative effects of hAEC transplantation on hepatic fibrosis, our study and others investigating stem cells were carried out predominantly in models of acute or short-term inflammation in which primarily mild fibrosis was evident [5], [6], [13]. Therefore, the effects of cellular therapy in models of chronic inflammation with well established fibrosis, which better reflect the clinical problem of advanced liver disease and cirrhosis, remain uncertain. Furthermore, there is no data on the efficacy of an additional cell dose or the generation of antibodies against the transplanted cells which may influence the timing and donor selection for subsequent treatments. Thus, using mice chronically injured with long-term CCl_4_ treatment, we investigated the efficacy of a single versus double hAEC dose and the effect of transplanted hAEC on host T cells and anti-hAEC antibody generation. In order to gain an understanding of potential anti-fibrotic mechanisms, we studied the effects of hAEC transplantation on hepatic macrophages that play a pivotal role in mediating fibrogenesis and fibrosis resolution [18], [19].

## Materials and Methods

### Ethics Statement

The study was approved by the Southern Health and Royal Women’s Hospital Human Research Ethics Committees and Monash University and University of Melbourne Institutional Review Boards. Informed, written consent was obtained from healthy women with a normal singleton pregnancy prior to elective cesarean section at term (37–40 weeks gestation; n = 16). Six week old male C57BL/6 mice were purchased from Monash Animal Services, Melbourne, Australia. Experimentation on C57BL/6 mice was approved by the Animal Ethics Committee, Monash University (approval number MMCB 2008/17).

### Isolation of hAEC

hAEC were isolated from amnion membranes and purity assessed as described previously [20]. Briefly, amnion membranes were cut into small pieces and digested twice in 0.05% trypsin:EDTA (Gibco, Grand Island, NY) for 40 min at 37°C. Following inactivation of trypsin with newborn calf serum, dispersed cells were washed in DMEM/F12 medium (Gibco) and erythrocytes lysed in hypotonic solution. Batches ≥99% positive for the epithelial markers cytokeratin-7 and 8/18 (Dako, Glostrup, Denmark) by flow cytometry and displaying a cobblestone epithelial morphology in culture [21], were transplanted into C57BL/6 mice (Figure S1).

### hAEC-splenocyte Co-culture

hAEC have been shown to suppress T cell proliferation in hAEC-splenocyte co-culture assays [17], [22]. To explore whether apoptosis played a role, hAEC were co-cultured with murine splenocytes stimulated with the mitogen Concanavalin A. The CD45+/CD3+/Annexin V+ cell population was determined by flow cytometry. Primary antibodies were linked to PE, Cy7 and FITC (BD Biosciences) and diluted 1∶200, 1∶50 and 1∶20, respectively.

### Murine Hepatic Stellate Cell (HSC) Isolation and Atreatment

HSC were isolated as described previously [23]. Briefly livers were perfused and digested with collagenase and pronase. HSC were isolated by Nycodenz density gradient centrifugation and purity was determined by vitamin A autofluorescence. To examine the effects of factors secreted by hAEC on transforming growth factor beta-1 (TGFβ-1) and collagen, murine HSC were grown in 24 well plates and stimulated with 25% hAEC conditioned media for 48 h. TGFβ-1 was measured by ELISA and collagen synthesis by the ^3^H-proline incorporation assay. HSC were washed, incubated on ice with trichloroacetic acid and sodium hydroxide added. Equal volumes of lysate and hydrochloric acid were added to 10 parts scintillation fluid and the beta emission was measured.

### Induction of Hepatic Fibrosis and hAEC Transplantation

Intraperitoneal injections of CCl_4_ (Merck, Darmstadt, Germany; 1 µl/g body weight diluted 1∶10 in olive oil) were administered to mice twice weekly for 12 weeks. hAEC (2×10^6^) were injected via the tail vein at week 8 (single dose group) or at weeks 8 and 10 (double dose group) of CCl_4_ treatment. The number of hAEC infused was based on previous experiments to identify the optimal dose [13]. Control groups consisted of mice given CCl_4_ only and healthy, untreated mice. Each cohort consisted of n = 8 mice. The animals were culled at 12 weeks and blood and liver tissue collected.

### Detection of Anti-hAEC Antibodies in Murine Serum

hAEC (2×10^6^; n = 4) were injected into healthy C57BL/6 mice (n = 4) and blood collected two weeks later (single cell dose group). Cell injection was repeated immediately after the first blood collection and blood collected again after a further two weeks (double cell dose group). To determine if there were anti-hAEC antibodies, the murine serum was diluted 1∶100 and mixed with suspensions of the same batches of hAEC that had been injected previously. The hAEC and serum were incubated for 30 min to enable anti-hAEC antibodies present in the murine serum to bind to the cells. Following three washes, the hAEC were incubated for 30 min with rabbit anti-mouse AlexaFluro-488 secondary antibody (1∶100; Molecular Probes, Eugene, OR). Cells were washed thoroughly and analysed by flow cytometry. hAEC alone and cells incubated with serum from healthy, non-injected mice served as negative controls.

### Immunohistochemistry

Mouse liver tissue was fixed in 10% neutral buffered formalin. Paraffin sections, 4 µm in thickness were dewaxed and rehydrated. hAEC present in the liver were identified by detecting human inner mitochondrial membrane (IMM; 1∶100; Millipore, Billerica, MA) protein and human leukocyte antigen (HLA)-G (1∶50; BD Biosciences, San Jose, CA) on serial sections following antigen retrieval in 0.01 M citrate buffer [13]. To examine if hAEC in the liver displayed features of mature hepatocytes, serial sections were stained for human albumin (1∶5000; Abcam, Cambridge, UK) and hepatic nuclear factor 4 alpha (HNF4α; 1∶150; Cell Signalling Technology, Danvers, MA) after antigen retrieval in citrate buffer. Hepatic macrophages were identified by staining with F4/80 (1∶250; a gift from Dr Richard Kitching, Monash University); T cells with biotinylated CD3 (1∶200; BD Biosciences), CD4 and CD8 (1∶100 and 1∶50, respectively; from Dr R Kitching) and activated HSC or myofibroblasts using α-smooth muscle actin (α-SMA; 1∶5000; Sigma-Aldrich, St Louis, MO).

Briefly, endogenous peroxidase activity was quenched by adding 0.3–3% H_2_O_2_ (IMM, HLA-G, F4/80, albumin, CD4, CD8, HNF4α) or peroxidase block (Dako; α-SMA, CD3). Non-specific binding was minimized with CAS protein blocking solution (Invitrogen, Camarillo, CA). Primary antibodies were incubated at room temperature for 30 min (α-SMA) or overnight at 4°C. Primary antibodies were either omitted from negative controls (F4/80, albumin, HNF4α) or incubated with isotype matched IgG (α-SMA) or pre-immune serum (IMM, HLA-G, CD3, CD4, CD8). After several washes sections were incubated with biotinylated rabbit anti-mouse IgG2a (Invitrogen; 1∶300; α-SMA, IMM, HLA-G, CD3), goat anti-rabbit IgG (Vector Laboratories, Burlingame, CA; 1∶200; albumin, HNF4α) or rabbit anti-rat IgG (Dako; 1∶150; F4/80, CD4, CD8). Antibody binding was detected using ABC kit reagents (Vector Laboratories) followed by DAB chromogen (Sigma-Aldrich). Slides were lightly counterstained with hematoxylin and mounted in DPX.

### Picrosirius Red Staining and Computer-assisted Morphometry

To identify the extent of histological fibrosis, 4 µm thick deparaffinized liver sections were stained as previously described [13]. Briefly, sections were incubated in picrosirius red (Sigma-Aldrich; Direct Red 80, 0.1% wt/vol in saturated picric acid) for 90 min and washed with acetic acid and water (1∶200). Fifteen non-overlapping fields were acquired, images digitized and fibrosis area measured by computer-assisted morphometry using Scion Image for Windows (vAlpha 4.0.3.2, Scion Corporation, Frederick, MD).

### Enzyme-linked Immunosorbent Assays (ELISA)

The concentrations of TGFβ-1, fractalkine (CX3CL1) and monocyte chemoattractant protein-1 (MCP-1) in liver tissue were measured by ELISA. Snap frozen liver tissue (∼150 mg) was homogenized in lysis buffer (50 mM Tris-HCl, 150 mM NaCl, 1 mM EDTA, 1% Triton X-100, 0.5% Tween-20, 0.1% SDS) containing a protease inhibitor cocktail (Roche, Mannheim, Germany). Lysates were sonicated and centrifuged at 14,000 g for 15 min at 4°C and supernatants collected. For TGFβ-1 measurement samples were activated with acetic acid/urea. ELISA plates were coated with antibodies against murine TGFβ-1 and CX3CL1 (R&D Systems, Minneapolis, MN) and murine MCP-1 (BD Pharmingen, San Diego, CA). Tissue lysates or media from murine HSC cultures were added and plates incubated for 2 h at room temperature. After several washes detection reagents were added and absorbencies read on a microplate reader (Magellan, Tecan, Austria). The concentrations were calculated from the standard curve generated by the plate reader software. The data was normalized against total protein concentrations that were measured using the BCA assay (Thermo Scientific, Rockford, IL).

### Real Time Quantitative Polymerase Chain Reaction (RT-qPCR)

The expression of M1 and M2 associated macrophage genes and matrix metalloproteinase (MMP) genes was studied by RT-qPCR. Total RNA was isolated from snap frozen liver tissue using the RNeasy mini kit (Qiagen, Hilden, Germany). RNA was converted to cDNA using the High-Capacity Reverse Transcription Kit (Applied Biosystems, Foster City, CA) and amplified using Power Sybr Green (Applied Biosystems, Warrington, UK) on a Rotor Gene 3000 light cycler (Qiagen, Sydney, Australia). The nucleotide sequences of primers used in the PCR are listed in Table 1. Samples were denatured at 95°C for 15 sec and annealed/extended at 60°C for 1 min for 40 cycles. Data was normalized to GAPDH and fold change calculated by the ^ΔΔ^CT method using healthy mice as the calibrator.

**Table 1:** Primers used to analyze expression of M1 and M2 macrophage genes and MMP.

Gene	Forward 5′→3′	Reverse 5′→3′
**M1**		
CCL17	CGAGAGTGCTGCCTGGATTACT	GGTCTGCACAGATGAGCTTGCC
CCL5	CCTGCTGCTTTGCCTACCTCTC	ACACACTTGGCGGTTCCTTCGA
IL-12b	CTCAGAAGCTAACCATCTCCTGG	CACAGGTGAGGTTCACTGTTTC
**M2**		
Cnrip-1	CGGCATCTATGACACAGAAGGTG	GGCAATGGTCTCGCTTGTGGTA
CD206	GTTCACCTGGAGTGATGGTTCTC	AGGACATGCCAGGGTCACCTTT
IL-10	CGGGAAGACAATAACTGCACCC	CGGTTAGCAGTATGTTGTCCAGC
YM-1	CCAGCATATGGGCATACCTT	AGACCTCAGTGGCTCCTTCA
Arg-1	CATTGGCTTGCGAGACGTAGAC	GCTGAAGGTCTCTTCCATCACC
Cadm-1	ACTTCTGCCAGCTCTACACGGA	CCCTTCAACTGCCGTGTCTTTC
CD36	GGACATTGAGATTCTTTTCCTCTG	GCAAAGGCATTGGCTGGAAGAAC
**MMP**		
MMP-9	CTGGACAGCCAGACACTAAAG	CTCGCGGCAAGTCTTCAGAG
MMP-12	CATGAAGCGTGAGGATGTAGAC	TGGGCTAGTGTACCACCTTTG
**Housekeeping**		
GAPDH	TGTTCCTACCCCCAATGTGT	TGTGAGGGAGATGCTCAGTG

### Statistical Analysis

Data were analysed by one-way ANOVA with Tukey’s post hoc test for multiple comparisons. Planned comparisons of numbers of hAEC engrafted in the liver following a single and double dose were analysed by Student’s t test. Analyses were carried out using GraphPad Prism software (v5.0d for Mac OS X, San Diego, CA). P<0.05 was considered to be statistically significant. Data are shown as mean±SEM.

## Results

### Histological Liver Fibrosis Increases Over Time Following CCl_4_ Administration

The extent of histological fibrosis was determined in picrosirius red-stained liver tissue sections. Twice weekly intraperitoneal administration of CCl_4_ to wild type C57BL/6 mice over a period of 4–12 weeks resulted in a progressive increase in histological fibrosis. Minimal fibrosis was seen at 4 weeks while there was clear evidence of bridging fibrosis at 12 weeks (Figure S2A). Fibrosis area, determined by computer-assisted morphometry, increased by nearly 50% from 4 to 8 weeks of CCl_4_ treatment (mean values of 2.07% and 3.01% at 4 and 8 weeks, respectively) and to 3.9 and 4.25% fibrosis area by weeks 10 and 12, respectively (P<0.05 vs 4 weeks; Figure S2B). Based on these findings, hAEC were transplanted at week 8 of CCl_4_ administration to test the anti-fibrotic efficacy of a single dose. To test the effect of a second dose in advanced fibrosis, hAEC were injected into a separate group of mice at week 8 and again at week 10. CCl_4_ administration continued until week 12, when both treated and control animals were culled.

### hAEC Engraft in Mouse Liver and Decrease CCl_4_-induced T Cell Infiltration

**Figure 1:**
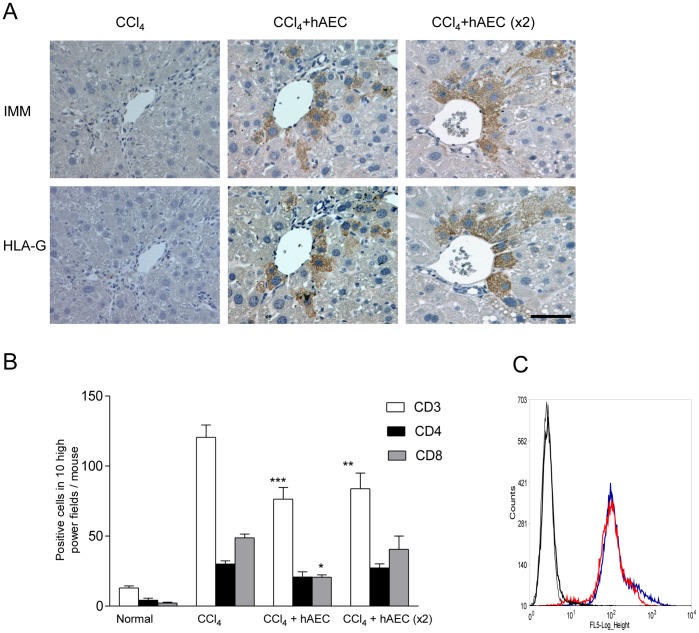
Engraftment of human amniotic epithelial cells (hAEC) in the liver and immune surveillance. A single or double hAEC dose was infused intravenously into C57BL/6 mice with established fibrosis induced by prolonged carbon tetrachloride (CCl_4_) treatment. Engrafted hAEC were identified by immunohistochemistry for human inner mitochondrial membrane (IMM) protein and HLA-G on serial tissue sections (A). The numbers of CD3 and CD8 positive T cells in the liver were significantly lower in mice treated with hAEC (B). Serum from mice injected with hAEC contained anti-hAEC antibodies that bound to hAEC and were detected by flow cytometry. Red and blue lines on the representative flow plot shown correspond to single and double hAEC infusions, respectively. Controls (grey lines) consisted of hAEC alone or hAEC reacted with serum from non-injected mice (C). Scale bar  = 50 µm (IMM/HLA-G). *, ** and ***  =  P<0.05, 0.01 and 0.001, respectively.

Intravenously infused hAEC were detected in liver tissue by immunohistochemistry for IMM protein and HLA-G. Intact, positively stained cells for both human markers were identified in serial tissue sections of animals given single and double hAEC doses (Figure 1A). hAEC were scattered throughout the hepatic acinus but were found predominantly in zone 1. These findings confirm that the infused hAEC had migrated, engrafted and remained in the injured liver for 2 to 4 weeks following cell transplantation. The number of IMM and HLA-G positive cells present in the liver increased with the second injection (mean ± SEM/high power microscopic field  = 3.03±0.41 and 4.83±0.52 for single and double doses, respectively; P<0.05). Infused hAEC express several hepatocyte specific genes including albumin but lack HNF4α, a key transcription factor present in mature hepatocytes [24]. Engrafted hAEC (HLA-G+ cells) were albumin positive and some were HNF4α+ (Figure S3), suggesting that some of the engrafted hAEC had differentiated into mature hepatocyte-like cells in the liver.

Given the presence of hAEC in mouse liver, we next determined whether there was an effect on T cells that could contribute to hepatic inflammation and to cell mediated rejection of the engrafted hAEC. We found an increase in hepatic CD3 positive T cells with CCl_4_ treatment, consistent with reports of elevated T cell numbers in liver inflammation and fibrosis [25], (Figure 1B). Interestingly, subsequent to the single and double hAEC doses, the number of CD3 positive cells was reduced (P<0.05 and 0.01 for CCl_4_ vs single and double doses, respectively). Dense cellular infiltration typically seen in cell-mediated graft rejection was not observed in the liver sections. Analysis of T cell sub-populations showed that the number of CD4+ cells remained unaltered whereas CD8+ cells were lower in hAEC treated mice (P<0.05 for CCl_4_ vs single dose; Figure 1B and Figure S4). We explored if hAEC induced apoptosis contributed to the overall reduction in T cell numbers. However, the CD45+/CD3+/annexin V+ population remained unaltered when murine splenocytes were co-cultured with hAEC (data not shown).

### Transplanted hAEC Elicit an Antibody Response

In separate experiments, we examined whether a humoral response with murine anti-hAEC antibodies was generated against the transplanted cells by analysing sera collected two weeks after healthy mice were given single and double hAEC doses. Similar levels of anti-hAEC antibodies were present in mice given single and double cell doses as shown by the mean fluorescence intensities of antibody bound hAEC analysed by flow cytometry (172.6±51.1 and 226.8±46 for single and double doses, respectively; P = 0.46, Figure 1C), indicating that a second hAEC infusion did not lead to further increases in circulating anti-hAEC antibodies.

### hAEC Transplantation Reduces Established Hepatic Fibrosis in CCl_4_ Treated Mice

**Figure 2:**
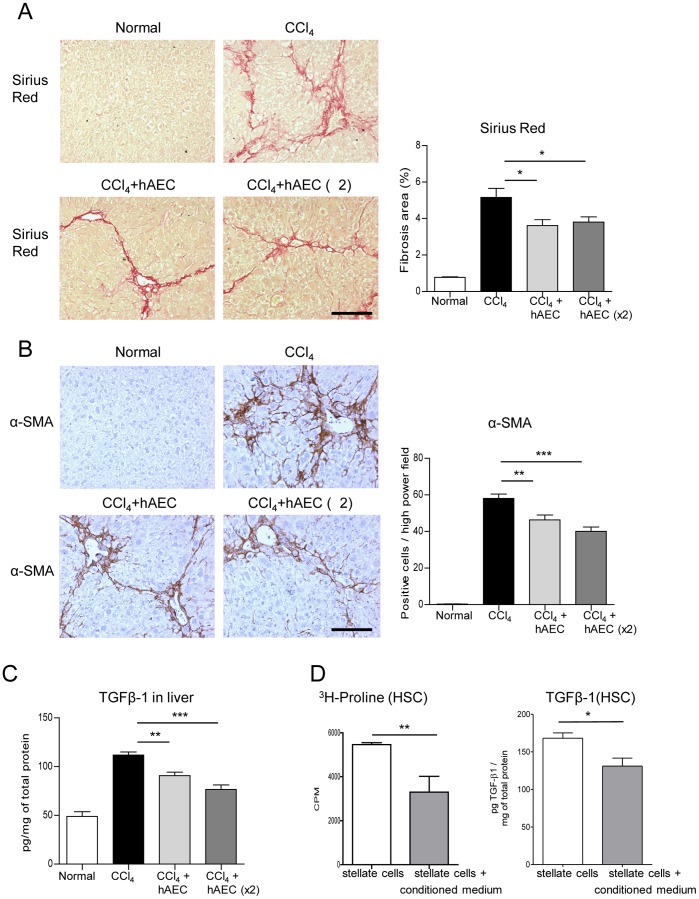
Human amniotic epithelial cell transplantation reduces established fibrosis. Sirius red staining of collagen and α-smooth muscle actin (α-SMA) immunohistochemistry of activated hepatic stellate cells (HSC) and quantitation are shown. C57BL/6 mice with established fibrosis induced by prolonged carbon tetrachloride (CCl_4_) administration were given a single or double hAEC dose. The hAEC-treated mice had significantly reduced fibrosis area (A) and numbers of activated HSC (B). The concentrations of the main pro-fibrogenic cytokine TGFβ-1 in the liver was measured by ELISA and found to be significantly lower in hAEC-treated mice (C). Murine HSC were treated with hAEC conditioned medium. TGFβ-1 and collagen synthesis declined significantly in HSC exposed to conditioned medium (D). Scale bars  = 100 µm. *, ** and *** P<0.05, 0.01 and 0.001, respectively.

Given the evidence of a humoral immune response against hAEC, we then examined whether the cells would be effective in reducing established hepatic fibrosis. Computer-assisted morphometry of picrosirius red-stained liver sections showed significantly less fibrosis area in CCl_4_-treated mice given hAEC compared to mice given CCl_4_ alone (P<0.05; Figure 2A). There was no further reduction in animals receiving a second hAEC dose. HSC are the principal collagen producing cell in the liver and when activated to a myofibroblast phenotype show increased expression of α-SMA. Mice given CCl_4_ plus hAEC showed fewer activated HSC compared to those given CCl_4_ alone (P<0.01; Figure 2B) with further reduction seen in mice receiving the double hAEC dose (P<0.001 compared to mice given CCl_4_ only). TGFβ-1 drives HSC activation and is thought to be the most potent pro-fibrogenic cytokine in the liver [26]. We measured TGFβ-1 protein in liver tissue lysates and found that mice given CCl_4_ plus hAEC had significantly less TGFβ-1 compared to mice given CCl_4_ alone (P<0.01; Figure 2C). TGFβ-1 levels declined further in mice treated with a double hAEC dose (P<0.001 compared to mice given CCl_4_ alone). We then explored if hAEC secreted factors acting directly on HSC could contribute to the reduction in hepatic fibrosis and TGFβ-1. Murine HSC were stimulated with media conditioned by hAEC. TGFβ-1 output and collagen synthesis declined significantly in the treated cultures (P = 0.016 and 0.003, respectively; Figure 2D).

### hAEC Transplantation Reduces Hepatic Macrophage Numbers and Induces an M2 Phenotype

**Figure 3:**
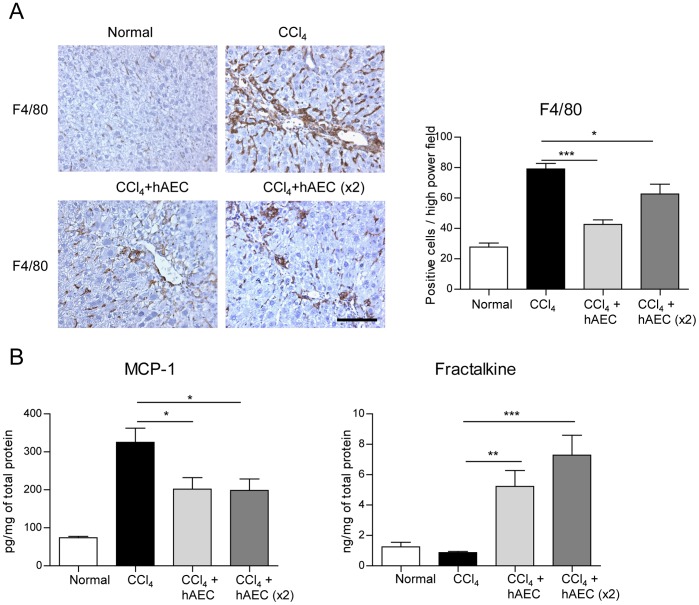
Human amniotic epithelial cell transplantation lowers hepatic macrophage number and alters monocyte recruitment factors. Hepatic macrophages were identified by F4/80 immunohistochemistry. C57BL/6 mice with established fibrosis treated with single or double hAEC doses had significantly lower number of F4/80 positive macrophages in the liver compared with animals given carbon tetrachloride (CCl_4_) only (A). The concentrations of monocyte recruitment factors measured by ELISA showed that hAEC treatment led to a significant reduction in MCP-1 and increased levels of fractalkine in the liver compared to CCl_4_ treated control animals (B). Scale bar  = 100 µm. *, ** and *** P<0.05, 0.01 and 0.001, respectively.

Macrophages play a pivotal role in hepatic fibrogenesis as well as fibrosis resolution depending on their phenotype and local environment [27], [28]. Previous studies have reported a large influx of bone marrow derived F4/80 positive monocytes in response to elevated levels of hepatic chemokines such as MCP-1 and fractalkine following injection of toxins such as CCl_4_ [25], [29], [30]. Consistent with these studies, the number of F4/80 positive macrophages was elevated in CCl_4_ treated animals compared to healthy mice (P<0.001; Figure 3A). However, the number of F4/80 positive cells was significantly lower in mice treated with hAEC (P<0.001 and 0.05 for single and double doses, respectively). To explore potential mechanisms that could lead to a reduction in macrophage numbers, we measured MCP-1 protein in mouse liver tissue lysates and found less MCP-1 in mice treated with hAEC (P<0.05 vs. CCl_4_ treated animals; Figure 3B). In contrast, hepatic fractalkine protein levels increased in mice treated with hAEC (P<0.01 and 0.001 for single and double hAEC doses, respectively).

**Figure 4:**
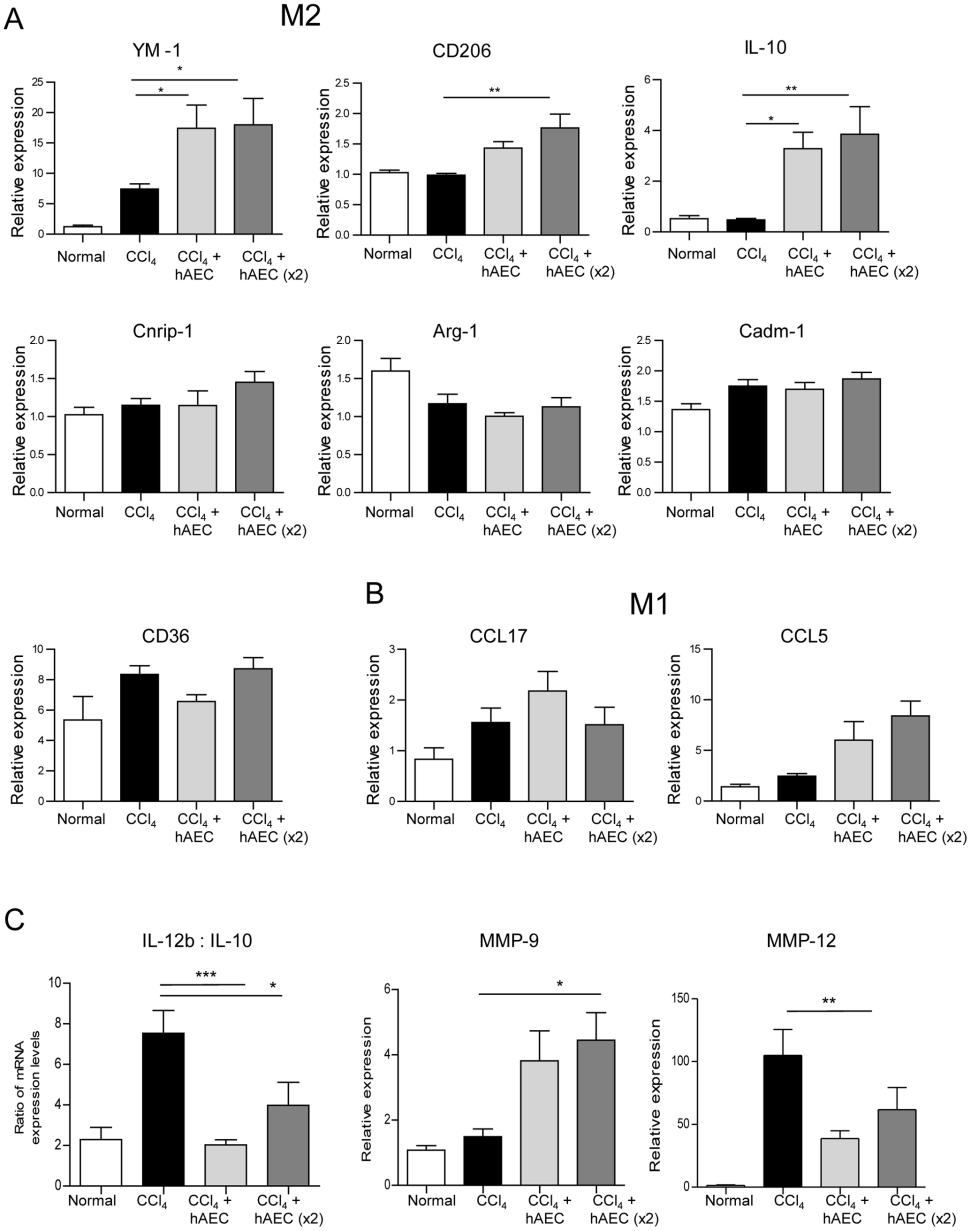
Human amniotic epithelial cell transplantation induces expression of genes associated with alternately activated (M2) macrophages. mRNA expression of classically (M1) and alternately activated M2 macrophage genes were analyzed by real time quantitative PCR and the fold change calculated by the ^ΔΔ^CT method. Expression of M2 associated YM-1, CD206 and IL-10 was significantly elevated in livers of mice treated with hAEC compared to animals given carbon tetrachloride (CCl_4_) alone (A). M1 associated genes CCL17 and CCL5 were expressed but levels did not alter with hAEC treatment (B). IL-12b:IL-10 ratio was skewed towards an M2 phenotype. MMP-9 increased and MMP-12 expression decreased with hAEC treatment (C). *, ** and *** P<0.05, 0.01 and 0.001, respectively.

The polarization of macrophages to a M2 alternatively activated phenotype is associated with hepatic wound healing [30]. We therefore examined the phenotype of the hepatic macrophage population by determining gene expression of known macrophage associated markers in whole liver extracts. We found that CCl_4_-treated mice which had been infused with hAEC had increased mRNA expression of M2 associated markers YM-1 (P<0.05), CD206 and IL-10 (P<0.01; Figure 4A) compared with mice given CCl_4_ alone. Cnrip-1 expression also increased in hAEC treated mice but did not reach significance while Arg-1 and Cadm-1 mRNA expression remained unaltered. We also examined the expression of classically activated M1 macrophages that have been linked to fibrosis progression [30]. We found no change in the expression of M1 associated markers CCL17 and CCL5 in hAEC treated mice compared with mice given CCl_4_ alone (Figure 4B). We also examined the ratio of IL-12b mRNA, which is expressed by M1 macrophages [31], to IL-10 mRNA to determine whether the macrophage phenotype was skewed towards M1 or M2. hAEC treated mice had increased IL-10 mRNA expression and a significant decrease in the IL-12b:IL-10 ratio consistent with skewing towards M2 macrophages (P<0.001 and <0.05 for single and double doses respectively; Figure 4C). Macrophages express MMP-9 and MMP-12, which are important regulators of fibrolysis [27], [31]. MMP-9 expression was elevated while MMP-12 decreased in hAEC treated mice (P<0.05; Figure 4C).

## Discussion

In this study we have shown that intravenously delivered human AEC engraft in injured livers of immunocompetent mice and lead to significant changes in hepatic macrophage numbers and phenotype and significantly reduce the extent of established fibrosis. hAEC engraftment was demonstrated by the presence of intact human IMM protein and HLA-G positive cells up to four weeks post transplantation. Similar outcomes showing grafted hAEC remaining several weeks after transplantation have also been reported in immunocompetent animals with brain, spinal cord and lung injury [32], [33], [34], [35]. Low levels of HLA Class IA expression, lack of co-stimulatory molecules CD80/86 and secreted factors such as TGF-β and IL-6 from hAEC that can suppress T cell expansion may have limited the surveillance of the engrafted cells by host T cells [14], [16], [22], [33]. However, anti-human antibodies were generated against the transplanted hAEC and it would be important to identify the antigens responsible, immunoglobulin sub-classes and the survival of hAEC following multiple infusions. Further, chemokines and adhesion molecules that regulate migration of intravenously infused hAEC to injury sites and subsequent engraftment remain uncertain.

The importance of our current findings in relation to human liver disease relates to the use of a model of established hepatic fibrosis and the evaluation of a second hAEC dose. We transplanted hAEC into mice only after advanced fibrosis was established and then continued CCl_4_ administration following cell transplantation to simulate the clinical scenario of advanced liver disease due to persistent liver injury such as that from chronic viral hepatitis or continued excessive alcohol use. Despite continuing liver injury from CCl_4_ exposure, hAEC transplantation resulted in a significant reduction in hepatic fibrosis area that was accompanied by a decrease in the number of α-SMA positive activated HSC. The decreased output of TGFβ-1, the principal pro-fibrogenic and regulatory cytokine for HSC activation, in livers of mice that received hAEC treatment may have led to lower numbers of collagen-producing HSC. This is supported by our *in vitro* finding that hAEC conditioned medium reduced TGFβ-1 and collagen synthesis by murine hepatic stellate cells. Mice receiving two hAEC infusions showed a marginal increase in the number of engrafted cells and a small decrease in HSC activation and TGFβ-1 protein levels compared to mice receiving a single infusion. However in this model, two hAEC infusions did not decrease fibrosis area to a greater extent than a single infusion. This finding may relate to the timing of the second hAEC dose with less time for engraftment (two weeks compared to four weeks after a single injection). Additionally, the number of cells infused or the route of cell administration may have been a factor in this outcome. Overall, these findings extend our previous observations [13] by demonstrating that hAEC transplantation is effective in reducing advanced hepatic fibrosis and supports the concept that hAEC should be further investigated in regard to optimal cell numbers for multiple transplantations, alternative routes of delivery (intrahepatic or intrasplenic) to test for maximal cell engraftment and long term outcomes in this and other animal models of chronic hepatic fibrosis.

We also investigated the potential role of macrophages in hepatic fibrosis reduction in this model. Consistent with previous studies, we found that CCl_4_-induced liver injury was associated with a significant increase in hepatic MCP-1, extensive infiltration of spindle-shaped F4/80 positive macrophages into the liver parenchyma and increased fibrosis compared to untreated controls [19], [36]. MCP-1 signalling via its receptor CCR2 on monocytes has been shown to induce the trafficking of these cells to sites of injury. Importantly, mice treated with hAEC had significantly lower levels of hepatic MCP-1 that correlated with reduced numbers of F4/80 positive macrophages and reduced fibrosis compared to mice receiving CCl_4_ alone. The importance of reduced MCP-1/CCR2 expression on fibrosis has been demonstrated in recent studies showing that blocking MCP-1 by RNA oligonucleotides significantly decreases CCl_4_-induced murine hepatic fibrosis [37], and that CCR2 knockout mice treated with CCl_4_ had significantly reduced hepatic fibrosis [36]. In contrast, we found that hepatic fractalkine (CX3CL1) protein levels increased significantly in hAEC treated mice compared to CCl_4_ alone. Increases in fractalkine have been shown to reduce HSC activation and fibrogenesis by inducing IL-10 and Arg-1 in hepatic macrophages [29]. Further, the interaction of fractalkine and its receptor, CX3CR1, leads to differentiation of macrophages having an anti-inflammatory phenotype (M2) and prolongs their survival leading to reduced fibrosis [38]. Our findings showing elevated IL-10 mRNA expression characteristic of alternately activated macrophages in livers of hAEC treated mice with increased levels of fractalkine lend support to this potential mechanism of fibrosis reduction.

The importance of macrophage plasticity in wound healing and hepatic tissue regeneration is increasingly recognised since alternatively activated M2 macrophages are thought to contribute to wound healing by secreting anti-inflammatory cytokines and collagen degrading enzymes [27], [30], [39]. Given the changes in F4/80 positive macrophage numbers and fibrosis with hAEC treatment, we explored whether the macrophage population displayed a phenotype consistent with wound healing and tissue remodelling. M2 macrophages express several distinct genes including YM-1, Cadm-1, CD206, CD36 and Cnrip-1. Analysis of hepatic mRNA expression showed that YM-1 mRNA, a chitinase family member, the anti-inflammatory cytokine IL-10 and the mannose receptor CD206 produced by alternatively activated macrophages, were significantly increased in CCl_4_-treated mice following hAEC transplantation compared to control animals given CCl_4_ alone. A decrease in the ratio of IL-12b to IL-10 expression further supports skewing towards a M2 phenotype. In addition, macrophages are an important source of matrix degrading enzymes. Liver from hAEC treated mice demonstrated significantly greater expression of MMP-9, which effectively degrades collagen, and significantly less MMP-12, which has been reported to inhibit the production of MMP-9 and increase hepatic fibrosis [40]. These are important new findings as there are few reports describing the effects of exogenous stem cells on macrophage phenotype. Mesenchymal stem cells have very recently been shown to regulate the switching of macrophages to a M2 phenotype in a murine model of myocardial infarction [41] and multipotent adult progenitor cells to induce a beneficial shift from M1 to M2 in a rat model of spinal cord injury [42]. Recently a therapeutic avenue using M2 macrophages has been suggested as CCl_4_-treated mice infused with alternatively activated macrophages showed reduced hepatic fibrosis [39]. Therefore, it will be important to determine the influence of exogenous stem cells on hepatic macrophages in the setting of liver injury.

In summary, these findings suggest that hAEC induce changes to macrophage recruitment and promote a wound-healing phenotype that is associated with amelioration of hepatic fibrosis. Our results offer novel insights into potential mechanisms underlying the resolution of established hepatic fibrosis induced by exogenously delivered stem-like cells derived from the human placenta.

## Supporting Information

Figure S1 **Cytokeratin staining of human amniotic epithelial cells.** Isolated hAEC show positive staining for cytokeratin (CK)-7 and CK8/18 by flow cytometry and immunocytochemistry. Scale bar = 100 µm.
(TIF)

Figure S2 **The extent of hepatic fibrosis following long term carbon tetrachloride administration.** Sirius red stained collagen in liver tissue sections obtained from immunocompetent C57BL/6 mice given twice weekly injections of CCl_4_ showed increased scarring with bridging fibrosis after 10–12 weeks (A). Quantitative computer assisted morphometry of Sirius red stained fibrosis area reflects the significant increase in collagen deposition with prolonged CCl_4_ administration (B). Scale bar  = 200 µm. * P<0.05.
(TIF)

Figure S3 **Human amniotic epithelial cells in liver show features of hepatocytes.** HLA-G positive hAEC stained for albumin. Some of these cells were also positive for HNF4α. (+VE  =  positive, -VE  =  negative; Scale bar  = 50 µm).
(TIF)

Figure S4 **T cell populations in liver of human amniotic epithelial cell treated mice.** CD3, CD4 and CD8 populations in liver of hAEC treated and control groups of mice are shown. Scale bar = 50 µm.
(TIF)

## References

[1] Friedman SL (2008). Mechanisms of hepatic fibrogenesis.. Gastroenterology.

[2] Friedman LS (2010). Surgery in the patient with liver disease.. Trans Am Clin Climatol Assoc 121: 192–204; discussion 205.

[3] Hanje AJ, Patel T (2007). Preoperative evaluation of patients with liver disease.. Nat Clin Pract Gastroenterol Hepatol.

[4] Gilchrist ES, Plevris JN (2010). Bone marrow-derived stem cells in liver repair: 10 years down the line.. Liver Transpl.

[5] Tsai PC, Fu TW, Chen YM, Ko TL, Chen TH (2009). The therapeutic potential of human umbilical mesenchymal stem cells from Wharton’s jelly in the treatment of rat liver fibrosis.. Liver Transpl.

[6] Sakaida I, Terai S, Yamamoto N, Aoyama K, Ishikawa T (2004). Transplantation of bone marrow cells reduces CCl4-induced liver fibrosis in mice.. Hepatology.

[7] Fang B, Shi M, Liao L, Yang S, Liu Y (2004). Systemic infusion of FLK1(+) mesenchymal stem cells ameliorate carbon tetrachloride-induced liver fibrosis in mice.. Transplantation.

[8] Couto BG, Goldenberg RC, da Fonseca LM, Thomas J, Gutfilen B (2011). Bone marrow mononuclear cell therapy for patients with cirrhosis: a Phase 1 study.. Liver Int.

[9] Kharaziha P, Hellstrom PM, Noorinayer B, Farzaneh F, Aghajani K (2009). Improvement of liver function in liver cirrhosis patients after autologous mesenchymal stem cell injection: a phase I-II clinical trial.. Eur J Gastroenterol Hepatol.

[10] Pai M, Zacharoulis D, Milicevic MN, Helmy S, Jiao LR (2008). Autologous infusion of expanded mobilized adult bone marrow-derived CD34+ cells into patients with alcoholic liver cirrhosis.. Am J Gastroenterol.

[11] Levicar N, Pai M, Habib NA, Tait P, Jiao LR (2008). Long-term clinical results of autologous infusion of mobilized adult bone marrow derived CD34+ cells in patients with chronic liver disease.. Cell Prolif.

[12] Stutchfield BM, Forbes SJ, Wigmore SJ (2010). Prospects for stem cell transplantation in the treatment of hepatic disease.. Liver Transpl.

[13] Manuelpillai U, Tchongue J, Lourensz D, Vaghjiani V, Samuel CS (2010). Transplantation of human amnion epithelial cells reduces hepatic fibrosis in immunocompetent CCl-treated mice.. Cell Transplant.

[14] Manuelpillai U, Moodley Y, Borlongan CV, Parolini O (2011). Amniotic membrane and amniotic cells: Potential therapeutic tools to combat tissue inflammation and fibrosis?. Placenta.

[15] Ilancheran S, Moodley Y, Manuelpillai U (2009). Human fetal membranes: a source of stem cells for tissue regeneration and repair?. Placenta.

[16] Parolini O, Alviano F, Bagnara GP, Bilic G, Buhring HJ (2008). Concise review: isolation and characterization of cells from human term placenta: outcome of the first international Workshop on Placenta Derived Stem Cells.. Stem Cells.

[17] Wolbank S, Peterbauer A, Fahrner M, Hennerbichler S, van Griensven M (2007). Dose-dependent immunomodulatory effect of human stem cells from amniotic membrane: a comparison with human mesenchymal stem cells from adipose tissue.. Tissue Eng.

[18] Ramachandran P, Iredale JP (2009). Reversibility of liver fibrosis.. Ann Hepatol.

[19] Karlmark KR, Wasmuth HE, Trautwein C, Tacke F (2008). Chemokine-directed immune cell infiltration in acute and chronic liver disease.. Expert Rev Gastroenterol Hepatol.

[20] Miki T, Marongiu F, Dorko K, Ellis EC, Strom SC (2007). Isolation of amniotic epithelial stem cells.. Curr Protoc Stem Cell Biol Chapter 1: Unit 1E 3.

[21] Ilancheran S, Michalska A, Peh G, Wallace EM, Pera M (2007). Stem cells derived from human fetal membranes display multilineage differentiation potential.. Biol Reprod.

[22] Pratama G, Vaghjiani V, Tee JY, Liu YH, Tan C (2011). Changes in culture expanded human amniotic epithelial cells: implications for potential therapeutic applications.. PLoS One.

[23] Patella S PD, Tchongue J, de Kretser DM, Sievert W (2006). Follistatin attenuates early liver fibrosis: effects of hepatic stellate cell activation and hepatocyte apoptosis.. American Journal of Physiology Gastrointestinal and Liver Physiology.

[24] Takashima S, Ise H, Zhao P, Akaike T, Nikaido T (2004). Human amniotic epithelial cells possess hepatocyte-like characteristics and functions.. Cell Struct Funct.

[25] Wasmuth HE, Tacke F, Trautwein C (2010). Chemokines in liver inflammation and fibrosis.. Semin Liver Dis.

[26] Kisseleva T, Brenner DA (2007). Role of hepatic stellate cells in fibrogenesis and the reversal of fibrosis.. J Gastroenterol Hepatol.

[27] Wynn TA, Barron L (2010). Macrophages: master regulators of inflammation and fibrosis.. Semin Liver Dis.

[28] Kolios G, Valatas V, Kouroumalis E (2006). Role of Kupffer cells in the pathogenesis of liver disease.. World J Gastroenterol.

[29] Aoyama T, Inokuchi S, Brenner DA, Seki E (2010). CX3CL1-CX3CR1 interaction prevents carbon tetrachloride-induced liver inflammation and fibrosis in mice.. Hepatology.

[30] Heymann F, Trautwein C, Tacke F (2009). Monocytes and macrophages as cellular targets in liver fibrosis.. Inflamm Allergy Drug Targets.

[31] Murray PJ, Wynn TA (2011). Protective and pathogenic functions of macrophage subsets.. Nature reviews Immunology.

[32] Yang X, Song L, Wu N, Liu Z, Xue S (2010). An experimental study on intracerebroventricular transplantation of human amniotic epithelial cells in a rat model of Parkinson’s disease.. Neurol Res.

[33] Moodley Y, Ilancheran S, Samuel C, Vaghjiani V, Atienza D (2010). Human amnion epithelial cell transplantation abrogates lung fibrosis and augments repair.. Am J Respir Crit Care Med.

[34] Cargnoni A, Gibelli L, Tosini A, Signoroni PB, Nassuato C (2009). Transplantation of allogeneic and xenogeneic placenta-derived cells reduces bleomycin-induced lung fibrosis.. Cell Transplant.

[35] Liu T, Wu J, Huang Q, Hou Y, Jiang Z (2008). Human amniotic epithelial cells ameliorate behavioral dysfunction and reduce infarct size in the rat middle cerebral artery occlusion model.. Shock.

[36] Seki E, de Minicis S, Inokuchi S, Taura K, Miyai K (2009). CCR2 promotes hepatic fibrosis in mice.. Hepatology.

[37] Baeck C, Wehr A, Karlmark KR, Heymann F, Vucur M (2011). Pharmacological inhibition of the chemokine CCL2 (MCP-1) diminishes liver macrophage infiltration and steatohepatitis in chronic hepatic injury.. Gut.

[38] Karlmark KR, Zimmermann HW, Roderburg C, Gassler N, Wasmuth HE (2010). The fractalkine receptor CXCR1 protects against liver fibrosis by controlling differentiation and survival of infiltrating hepatic monocytes.. Hepatology.

[39] Thomas JA, Pope C, Wojtacha D, Robson AJ, Gordon-Walker TT (2011). Macrophage therapy for murine liver fibrosis recruits host effector cells improving fibrosis, regeneration, and function.. Hepatology.

[40] Madala SK, Pesce JT, Ramalingam TR, Wilson MS, Minnicozzi S (2010). Matrix metalloproteinase 12-deficiency augments extracellular matrix degrading metalloproteinases and attenuates IL-13-dependent fibrosis.. Journal of Immunology.

[41] Dayan V, Yannarelli G, Billia F, Filomeno P, Wang XH (2011). Mesenchymal stromal cells mediate a switch to alternatively activated monocytes/macrophages after acute myocardial infarction.. Basic Res Cardiol.

[42] Busch SA, Hamilton JA, Horn KP, Cuascut FX, Cutrone R (2011). Multipotent adult progenitor cells prevent macrophage-mediated axonal dieback and promote regrowth after spinal cord injury.. J Neurosci.

