# Supplementary figures and images for: Human Amniotic Epithelial Cell Transplantation Induces Markers of Alternative Macrophage Activation and Reduces Established Hepatic Fibrosis
Source: PLoS One. 2012 Jun 14;7(6):e38631. doi: 10.1371/journal.pone.0038631 (PMC3375296; doi:10.1371/journal.pone.0038631)

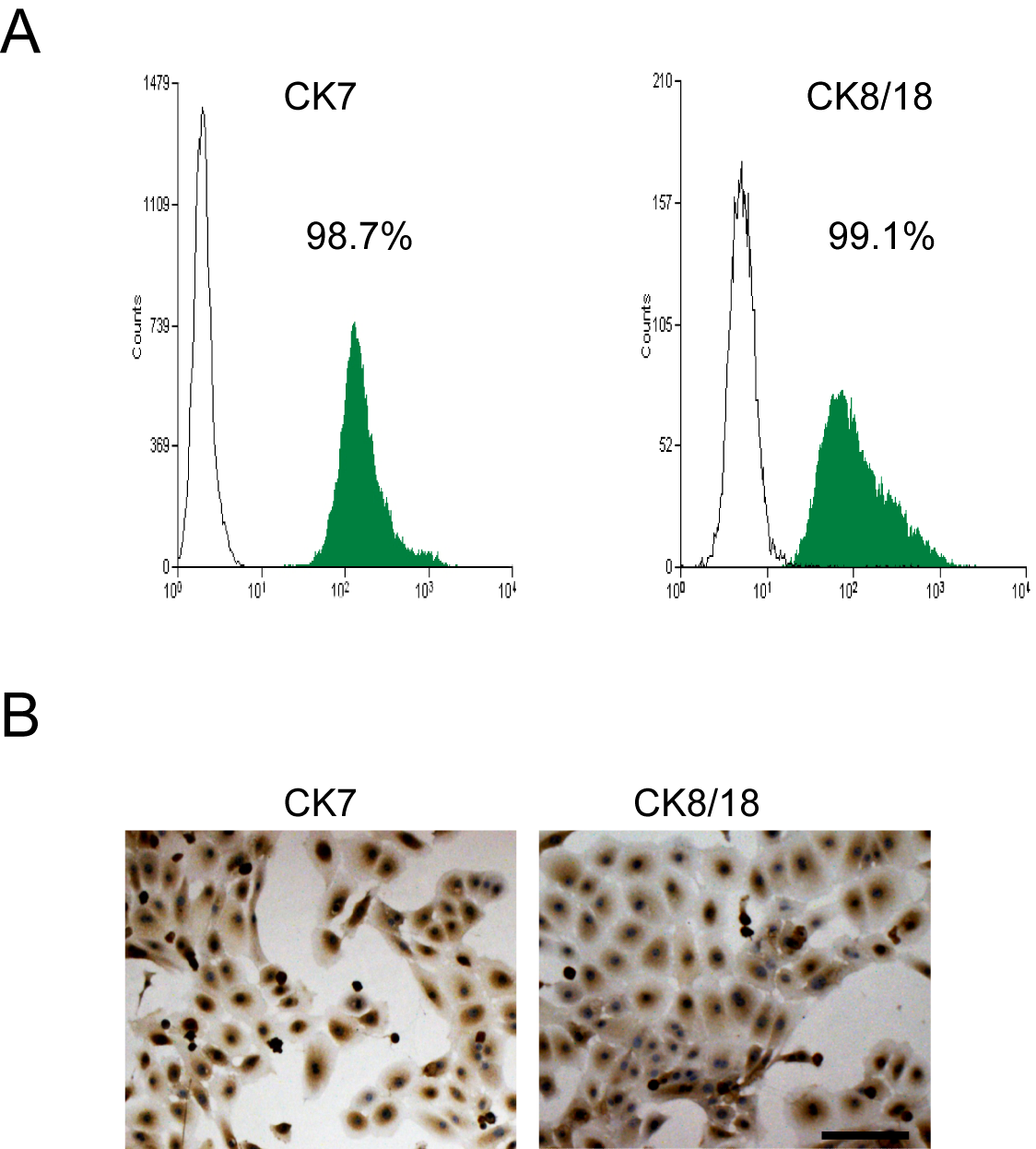

Supplement: Figure S1 — Cytokeratin staining of human amniotic epithelial cells. Isolated hAEC show positive staining for cytokeratin (CK)-7 and CK8/18 by flow cytometry and immunocytochemistry. Scale bar = 100 µm. (TIF) [file pone.0038631.s001.tif]

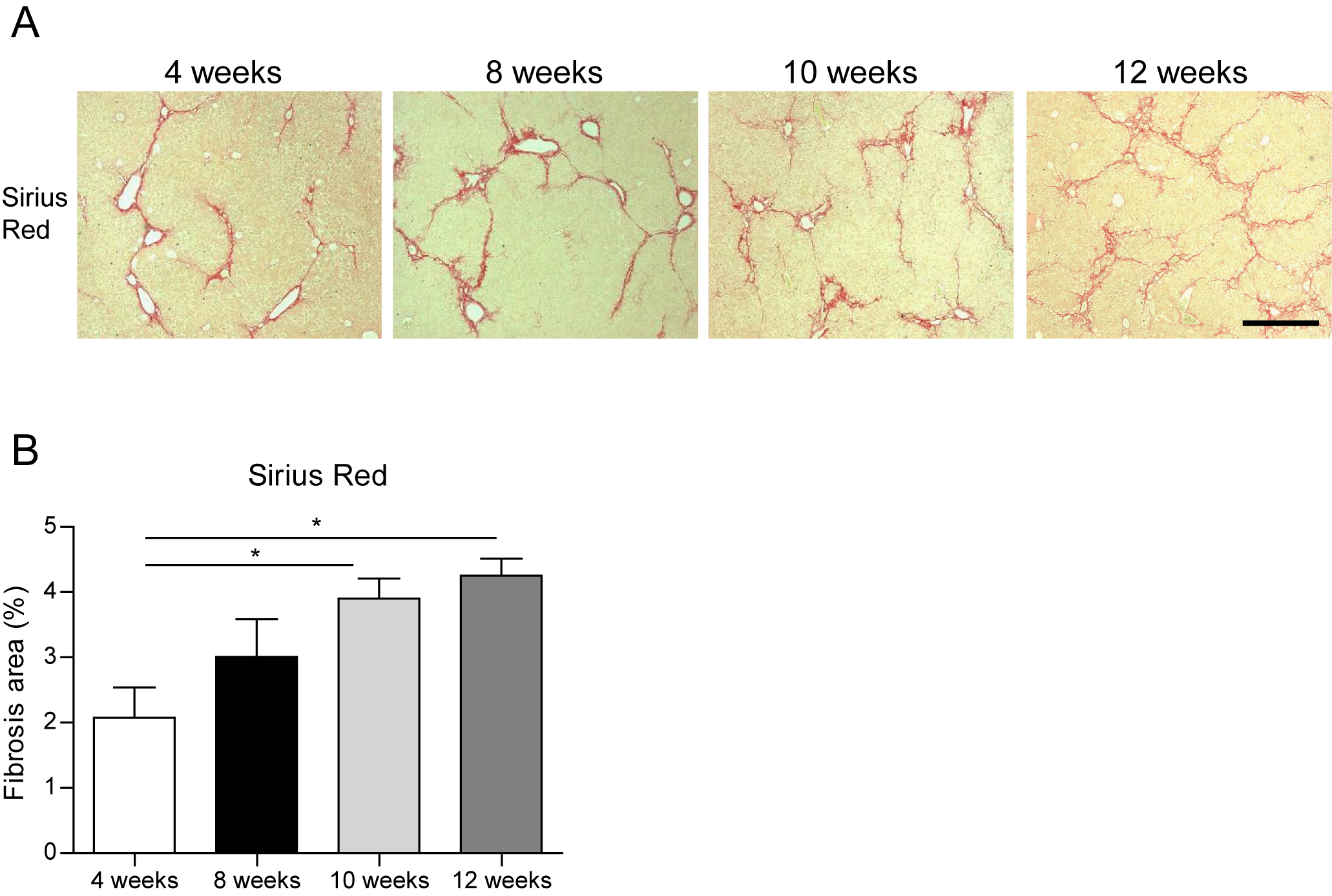

Supplement: Figure S2 — The extent of hepatic fibrosis following long term carbon tetrachloride administration. Sirius red stained collagen in liver tissue sections obtained from immunocompetent C57BL/6 mice given twice weekly injections of CCl4 showed increased scarring with bridging fibrosis after 10–12 weeks (A). Quantitative computer assisted morphometry of Sirius red stained fibrosis area reflects the significant increase in collagen deposition with prolonged CCl4 administration (B). Scale bar = 200 µm. * P<0.05. (TIF) [file pone.0038631.s002.tif]

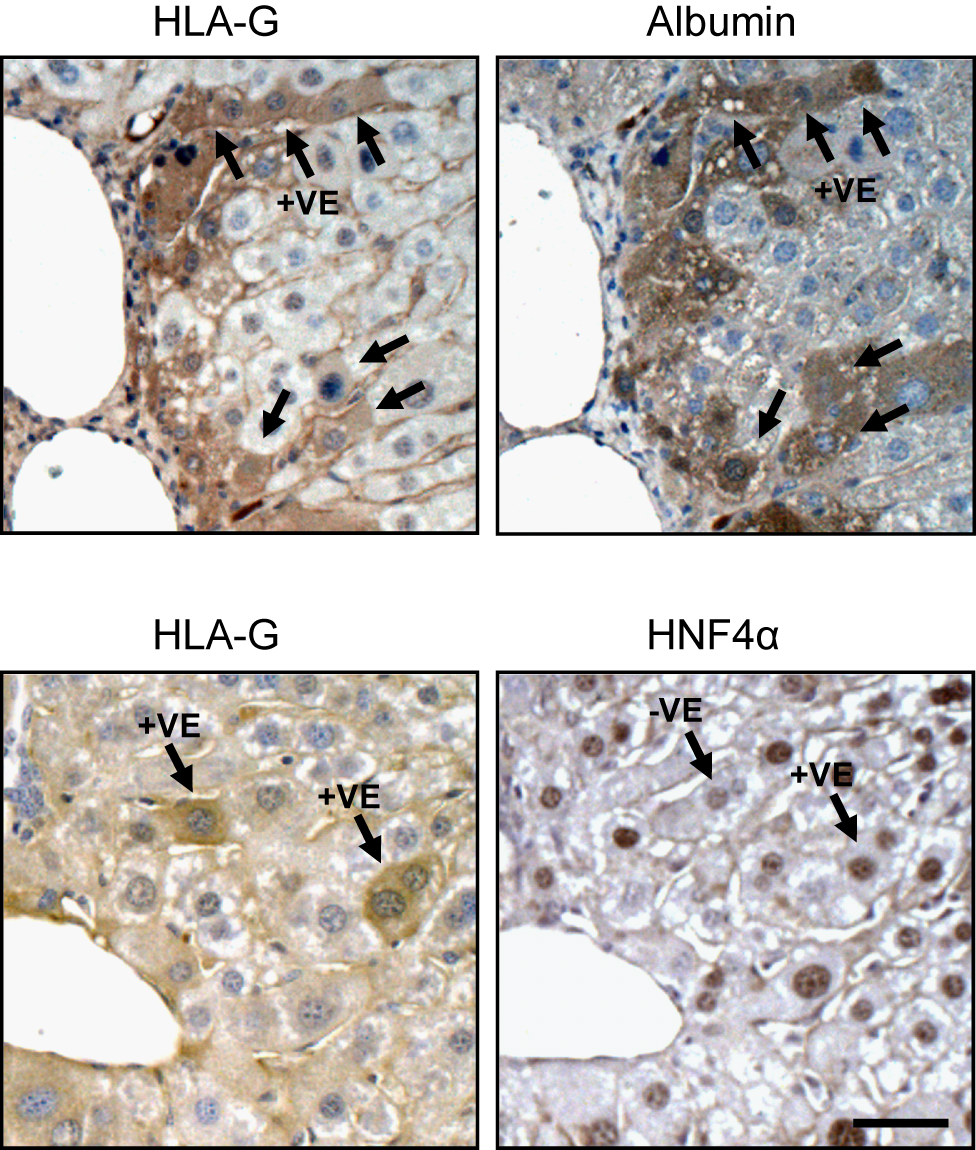

Supplement: Figure S3 — Human amniotic epithelial cells in liver show features of hepatocytes. HLA-G positive hAEC stained for albumin. Some of these cells were also positive for HNF4α. (+VE = positive, -VE = negative; Scale bar = 50 µm). (TIF) [file pone.0038631.s003.tif]

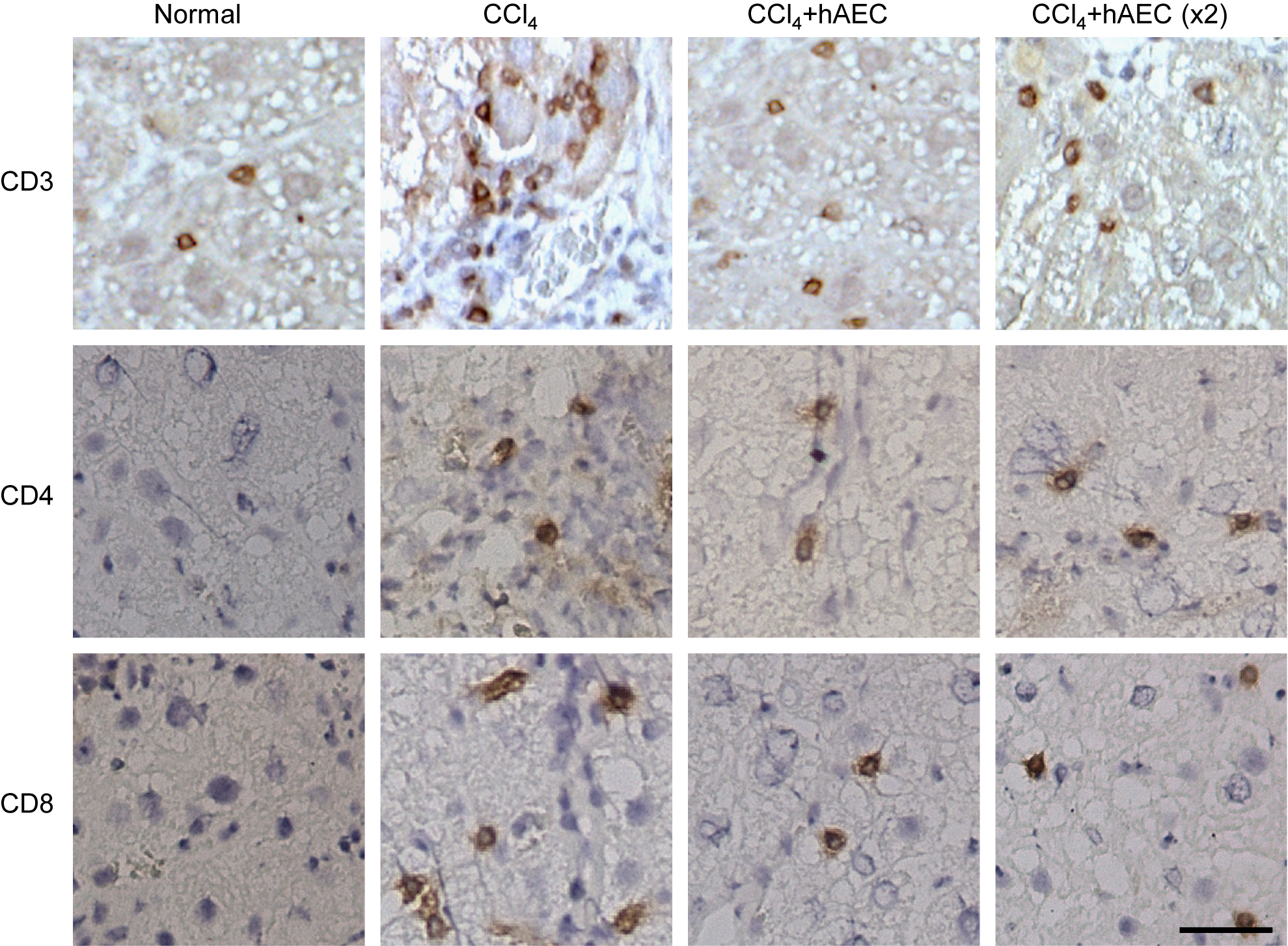

Supplement: Figure S4 — T cell populations in liver of human amniotic epithelial cell treated mice. CD3, CD4 and CD8 populations in liver of hAEC treated and control groups of mice are shown. Scale bar = 50 µm. (TIF) [file pone.0038631.s004.tif]
